# Do Epigenetic Marks Govern Bone Mass and Homeostasis?

**DOI:** 10.2174/138920212800543129

**Published:** 2012-05

**Authors:** Jesús Delgado-Calle, Pablo Garmilla, José A Riancho

**Affiliations:** Department of Internal Medicine, Hospital U.M. Valdecilla-IFIMAV-University of Cantabria, Santander, Spain

**Keywords:** DNA methylation, histones, miRNA, osteoblasts, osteoclasts, osteoporosis, gene expression.

## Abstract

Bone is a specialized connective tissue with a calcified extracellular matrix in which cells are embedded. Besides providing the internal support of the body and protection for vital organs, bone also has several important metabolic functions, especially in mineral homeostasis. Far from being a passive tissue, it is continuously being resorbed and formed again throughout life, by a process known as bone remodeling.

Bone development and remodeling are influenced by many factors, some of which may be modifiable in the early steps of life. Several studies have shown that environmental factors in uterus and in infancy may modify the skeletal growth pattern, influencing the risk of bone disease in later life. On the other hand, bone remodeling is a highly orchestrated multicellular process that requires the sequential and balanced events of osteoclast-mediated bone resorption and osteoblast-mediated bone formation. These processes are accompanied by specific gene expression patterns which are responsible for the differentiation of the mesenchymal and hematopoietic precursors of osteoblasts and osteoclasts, respectively, and the activity of differentiated bone cells. This review summarizes the current understanding of how epigenetic mechanisms influence these processes and their possible role in common skeletal diseases.

## INTRODUCTION

The social and public health measures implemented in the last century in developed countries have decreased the societal impact of communicable and other acute diseases to a great extent. Therefore, chronic disorders represent nowadays a major component of disease burden. Many of them belong to the group of the so-called “complex disorders”, which includes many prevalent disorders that are the final result of complex interactions of environmental and other acquired factors with genetic factors. Osteoporosis and osteoarthritis are the most prevalent skeletal disorders. In particular, osteoporosis has been estimated to affect about 30% of women and 12% of men above 50 years of age [1]. Osteoporosis is characterized by low bone mass and microarchitectural changes of bone tissue that result in a decreased bone resistance and susceptibility to fracture. Fractures in osteoporotic patients thus occur after low-impact trauma in the peripheral skeleton, particularly at the hip, the wrist and the humerus. Vertebral fractures are also very common and may appear even without any trauma, just as a consequence of the daily activities. 

Osteoporosis is considered as a complex disorder. On the one hand, several acquired factors have been shown to be associated with decreased bone mass and/or increased risk of fractures, including low body weight, heavy alcohol and tobacco consumption, immobilization, corticoid use, etc. Nutritional factors are also important. Although some discrepant studies have been published, most researchers and clinicians agree that protein-energy malnutrition, low calcium intake and an inadequate supply of vitamin D increase the risk of osteoporosis. On the other hand, many epidemiological studies have provided strong evidence for a role of heredity in osteoporosis. In fact genetic factors have been estimated to account for 40-80% of bone mineral density (BMD) variance [2-4]. Although fractures depend not only on the intrinsic properties of bone, but also on other personal and environmental factors, including the propensity to fall, a hereditary component of fractures has also been demonstrated. Thus, in the Study of Osteoporosis Fractures, a maternal history of hip fracture doubled the risk of fracture and the increase in risk remained significant after adjustment for bone density [5]. In a meta-analysis of several cohorts, Kanis *et al*. estimated that a family history of hip fracture in parents increased the risk of hip fracture (relative risk 2.3) and all osteoporotic fractures (relative risk 1.5) [6]. 

BMD changes through the life time of an individual. It accumulates during the growth period, reaches a peak by the third decade of life and then remains stable for some time. In later years, BMD begins to decrease progressively with aging. Therefore, osteoporosis may result from an inadequate peak bone mass attained in the early adulthood, from an accelerated loss of bone thereafter, or from a combination of both. The relative importance of peak bone mass and later losses on the development of osteoporosis probably varies among individuals, but the former is likely to be the most important. In fact, a 10% increase in peak BMD is predicted to delay the development of osteoporosis by 13 years, while a 10% change in the age at menopause or the rate of post-menopausal bone loss is predicted to delay osteoporosis by approximately 2 years, suggesting that peak BMD may be the single most important factor in the development of osteoporosis [7].

Theoretically, it could be anticipated that genetic factors have a more important influence on BMD in young individuals than in the elderly, as the relative contribution of environmental factors is likely stronger in the latter. Some studies support this notion. For example, Brown *et al*. studied 570 women from large Amish families and estimated that genes explained 58-88% of total variation in BMD in premenopausal women and 37-54% in the postmenopausal ones [8]. Likewise, in a twin study in Sweden, Michaelsson [9] estimated a greater heritability for hip fractures before the age of 69 years (0.68; 95% CI, 0.41-0.78) and between 69 and 79 years (0.47; 95% CI, 0.04-0.62) than for hip fractures after 79 years of age (0.03; 95% CI, 0.00-0.26). 

From the studies mentioned above, and many others, the importance of genetic factors in osteoporosis is out of doubt. Some rare cases of osteoporosis due to single-gene mutations have been identified. They include mutations of the lipoprotein receptor related protein 5 (LRP5), collagen, aromatase and estrogen receptor genes [10]. However, most cases appear to be polygenic in nature, with multiple genes involved, each one having only a modest influence on the phenotype. In the past 15 years many linkage and association studies have been performed trying to identify the genes actually involved. Indeed, association signals have been reported and successfully replicated at various loci, using candidate gene or, more recently, genome-wide approaches. 

However, as it is the case for other complex disorders, these studies have been somewhat disappointing because the combination of all the gene variants identified explains only a very small fraction of the disease risk. This suggest that, at the cellular level, those genetic variants have only a modest impact on gene expression [11]. The reasons explaining the missing heritability are disputed, but they are likely to include complex gene-gene and gene-environmental interactions. Indeed, as already mentioned, some environmental influences are well recognized risk factors for osteoporosis. Since the genome is highly stable, in general environmental factors influence genome activity by mechanisms that do not involve DNA sequence modifications. Some of them may represent epigenetic mechanisms, which are heritable through generations or cell divisions. Thus, the epigenome of an individual is currently seen as the result of genetic factors, environmental influences and stochastic variations. 

There are still very scarce data about the actual role of epigenetic mechanisms in bone disorders. However, several emerging lines of evidence suggest that they may be important in the biology of bone cells and the pathogenesis of osteoporosis. This review will summarize the current knowledge about these subjects.

## EPIGENETIC REGULATION OF GENE EXPRESSION

The group of well-known epigenetic mechanisms include DNA methylation, chromatin (in particular, histone) modifications and miRNAs [12]. However, the group is likely to be expanded in the future. For instance, the roles for hydroxymethylcytosine residues and CpG island shores have been recently proposed [13,14]. Among epigenetic mechanisms, DNA methylation and histone modification modulate gene transcription, whereas miRNAs act at the post-transcriptional level. The most widely studied epigenetic mark is DNA methylation, whose role in tumurogenesis has been clearly established. In humans DNA methylation consists in the addition of a methyl group in cytosines that precede guanines (CpGs), process catalyzed by DNA methyl transferases, using S-adenosyl-methionine as donor of methyl groups [15]. Interestingly, there are CpG-enriched areas in many gene promoters and their surrounding regions, known as CpG islands [16]. DNA methylation at these sites is usually associated with silencing of gene transcription. The exact mechanisms by which DNA methylation marks inhibit gene transcription are not fully understood yet. However, some data suggest that it can directly impair the binding of essential transcriptional factors to their target sites. 

Histone modifications, including methylation, acetylation, phosporilation, SUMOylation and ubiquitination have been also extensively studied [17,18]. The complexity of the histone tail modifications depends not only on the type of chemical groups added, but also on the aminoacid residue modified and the number of groups added. For example methylation may be in three different forms: mono-, di-, or trimethyl for lysines and mono- or di- (asymmetric or symmetric) for arginines [17]. In general, histone modifications can be divided into those that correlate with activation of transcription (mainly acetylation and phosphorilation) and those that correlate with repression (methylation, ubiquitination and sumoylation) [19]. The term “histone code” is frequently used to describe a specific set of modifications for a given task. 

The first miRNA was discovered in the early 90s [20] and the number of identified miRNAs have not stopped growing since then. MiRNAs are non coding RNAs of about 22 nucleotides. Approximately 1500 different miRNA have been identified in humans so far, and the number will probably increase in the future years [21]. Interestingly, bioinformatic predictions indicate that miRNAs may be involved in the regulation of 60% of the coding genes [22]. These small RNAs are initially transcribed as long primary transcripts, and then undergo a specific cleavage driven by the Drosha and Dicer enzymes. The mature miRNA loss one strand, whereas the other, know as the complementary miRNA strand, is loaded into the RISC protein complex, which mediates the effect on gene expression [23]. miRNAs bind to the 3´or 5´UTR of mes-senger RNAs and induce mRNA cleavage or translational repression, depending on the degree of complementarity [24]. When there is extensive complementarity, RISC mediates the cleavage of the target mRNA, whereas in case of loose complementarity the complex will impair the advance of the ribosome complex repressing translation. 

Although each epigenetic mechanism by itself is capable of affecting gene expression, they also interact with each other in a cooperative manner, allowing the cells to activate or repress gene expression in a time and tissue-specific manner. It has been shown that DNA methylation and histone posttranslational modifications contribute to the establish-ment and maintenance of chromatin accessibility [25]. Indeed, methyl-CpG biding proteins, which recognize methylated CpGs, recruit HDACs to methylated DNA, promoting the gain of repressive marks in the histone tails [26]. Likewise, miRNAs may also influence DNA methylation or chromatin remodeling [27]. Furthermore, it has been recently suggested that the expression of certain miRNAs may be controlled by CpG methylation and histone modification [28,29].

## THE COMPLEX PROCESSES OF BONE FORMATION AND BONE REMODELING

Bone tissue consists in a heavily mineralized extracellular matrix, with collagen as the major protein component, and different cell types. Far from being a static organ, bone changes in structure and composition from birth to adulthood. Modeling and remodeling are the processes by which bone adapts to external influences. Bone modeling is responsible for the gain in skeletal mass and the changes in skeletal size and shape taking place during the growth period, whereas bone remodeling replaces old bone by new bone tissue, throughout life, and specifically in the adult skeleton, to maintain bone mass, repair bone microfractures and allow the adaptation to external physical requirements [30]. 

The most important cells in bone homeostasis belong to two separate families: the osteoclastic and the osteoblastic lineages. Osteoclasts derive from hematopoietic precursors and are responsible for bone resorption. On the other hand, osteoblasts derive from local mesenchymal precursors and are responsible for bone formation. There are several types of cells within the osteoblastic lineage, with different gene repertoires, shapes and functions. The bone forming osteoblasts are cuboidal cells that synthesize alkaline phosphatase, collagen and other constituents of the bone matrix. The osteocytes are stellated cells that derive from osteoblasts that become embedded in the cell matrix they have formed. Finally, the so-called lining cells are flat-shaped cells that cover the inactive bone surfaces. However, cells in the osteoblastic lineage not only form bone, but also have other roles in bone metabolism. They modulate the proliferation and differentiation of osteoclast precursors, and regulate osteoclast activity [31,32].

**Fig. (1):**
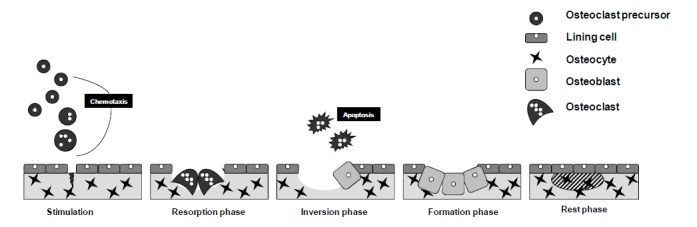
The bone remodeling cycle. The start of bone remodeling is probably driven by osteocytes (Stimulation). Osteoclasts are recruited to the bone surface by chemoatraction and remove a discrete packet of bone (Resorption). After a brief reversal phase in which osteoclast stop to resorbe bone and undergo apoptosis, osteoblasts arrive to the region and secrete bone matrix, filling the cavity (Formation). Note that some osteoblasts are buried within the new matrix, becoming osteocytes. The resorption phase last only a few weeks, whereas bone formation takes several months. Once the cavity is completely restored, bone enters the resting phase.

During bone modeling, resorption and bone formation occur on separates surfaces, whereas during remodeling, formation and resorption are coupled. The process of bone remodeling begins when a group of osteoclasts resorb a small volume of bone tissue (Fig. **1**). When this phase finishes, osteoblasts arrive to the area and fill with new bone the cavity eroded by osteoclasts. Therefore, the processes of bone resorption and bone formation are critical determinants of bone mass and strength. In fact, the decreased bone mass that characterizes osteoporosis at the tissue level represents the consequence of an imbalance between osteoclast and osteoblast function at the cellular level [33,34].

Maintaining skeletal properties and functionality depends on the organized action of all cell types present in this tissue. In fact, there are complex interaction networks between osteoblasts, osteoclasts and osteocytes. In particular, osteocytes are emerging as critical elements in the regulation of skeletal homeostasis. They may act as mechanosensors, mark the sites where a remodeling cycle must be initiated and secrete a number of factors that influence the activity of other cells in the osteoblastic as well as the osteoclastic lineages [35]. This interplay between cells promotes changes of the expression level of target genes, resulting in variations of cell activity. In addition, since bone remodeling takes place in a time and site-coordinated way, the differentiation of osteoblast and osteoclast precursors must also be controlled in a time and site-specific manner. The differentiation programs of these cells promote marked changes in gene expression, translated into different morphologies and activities, thus allowing the mature cells to achieve its expected function. Therefore, mechanisms regulating the transcriptional activity of those genes play a critical role in bone homeostasis and bone disease.

## EPIGENETIC MARKS IN THE OSTEOBLASTIC LINEAGE DIFFERENTIATION

**Fig. (2):**
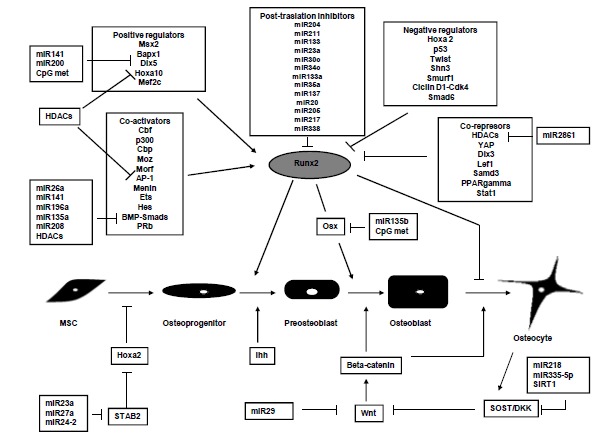
The osteoblastic lineage differentiation. A complex network drives the osteogenic differentiation of the mesenchymal stem cells (MSCs). Transcription factor Runx2 controls the process inducing the expression of key genes. Its expression is tightly regulated by a wide variety of mechanisms, including positive and negative modulators, co- activators and co-repressors, as well as epigenetic marks.

Osteoblasts and osteocytes originate from mesenchymal stem cells (MSCs). Interestingly, not only bone cells derive from these precursors, but also adipocytes and myogenic cells, as well as chondrocytes, share the same progenitor [36]. This highlights the need for mechanisms regulating lineage-specific differentiation of MSCs and then maintaining the mature phenotypes. Regarding the bone tissue, MSCs differentiate into osteoblasts and these will eventually evolve into osteocytes and lining cells, through a complex process that involves transcription factors and also modifications of the epigenetic marks (Fig. **2**) [37,38]. During osteogenic differentiation, MSCs undergo a dramatic transformation, at gene expression, functional and morphological levels [39]. Thus, cell shape changes from the polygonal bone forming osteoblasts to the dendrite-rich stellate osteocytes The podoplanin gene appears to be involved in this process, and its expression may be regulated by a cooperative crosstalk between DNA methylation and histone modification in osteoblastic cells [40]. On the other hand, the change in cell shape between bone forming osteoblasts and osteocytes is accompanied by different gene expression profiles. It is known that alkaline phosphatase activity, an enzyme critical for bone mineralization, is high in osteoblasts, the unique bone forming cell, whereas it is reduced in osteocytes, which do not produce bone [41]. In line with this, we recently demonstrated that osteoblasts and osteocytes have opposite DNA methylation profiles in the alkaline phosphatase (ALPL) promoter, which is hypomethylated in osteoblasts and hypermethylated in osteocytes, suggesting that DNA methylation is inhibiting ALPL expression in the latter ones [42]. The opposite is the case of SOST, which is actively expressed in osteocytes, but not in osteoblasts [39]. SOST is the gene encoding sclerostin, a peptide that tends to impair osteoblast activity by inhibiting Wnt signaling [43]. We have recently demonstrated that DNA methylation may be responsible for the repression of SOST expression in osteoblasts. Furthermore, we observed that DNA demethylation occurs during osteoblast-osteocyte transition, allowing osteocytes to express SOST [44]. In addition, Cohen-Kfir *et al*. suggested that sirtuin 1, a histone deacetylase, directly regulates SOST expression [45]. 

It has been shown that DNA demethylation induced by chemical compounds facilitates osteogenic gene expression and differentiation [46]. Likewise, it has been reported that reduced DNA methylation of other CpG islands in the promoter regions of osteocalcin (BGLAP) and osteopontin genes is associated with osteogenic differentiation [47,48]. On the other hand, high DNA methylation at the promoter of Brachyury transcription factor may be required for this process [49]. Other genes influencing osteogenesis, such as osterix, the osteogenic protein Dlx-5, aromatase and the estrogen receptor are also regulated by DNA methylation [50-53]. Not only DNA methylation is critical for osteogenic differentiation, but also chromatin remodeling plays an important role. It has been shown that different transcription factors induce chromatin remodeling at target promoters [54]. In fact, histone modifications are associated with BGLAP expression [55]. H3K4 and H3K6 methylation is associated with HOXa-10 and AP-2α expression respectively, and determine the advance of the osteogenic differentiation [56,57]. Other important genes regulated by histone modifications are Runx2, AP-1, ATF4 and Smads [58]. 

The analysis of miRNA arrays have identified several miRNAs whose expression changes during MSC differentiation, affecting target gene translation [59], and thus suggesting that miRNAs expression is actively involved in the regulation of this process (Table **1**). It has been shown that miRNAs regulate the expression of pivotal osteogenic transcription factors, such as Runx2 or Smads. Runx2 is required for determination of the osteoblast lineage. This transcription factor induces the differentiation of multipotent MSCs into immature osteoblasts modulating the expression of key genes during the early stages of osteoblast differentiation, such us collagen type 1 and 2, BGLAP, fibronectin, sclerostin or osteoprotegerin [60,61]. miR-204 and its homologue miR-211, as well as miR-133 and miR-135b, inhibit Runx2 [62-64]. On its turn, Runx2 may also regulate the expression of some miRNAs involved in the osteogenic process [65].

miRNAs are also actively involved in the regulation of Smad protein levels. Smads are intracellular proteins that transmit signals originating from the interaction of bone morphogenetic proteins (BMPs) and transforming growth factors (TGFs) with their receptors at the cell membrane. It is know that BMP/TGF and Runx2 pathways converge for the transcriptional control of bone formation. Thus, Smad proteins are recruited to Runx2 regulatory complexes and collaborate to modulate gene expression [66]. miR-26a has been shown to negatively regulate Smad1, resulting in a decreased expression of various bone markers, such as ALPL, BGLAP, osteopontin, and COL2A1 [67]. miR-135 regulates other member of the Smad family, Smad5 [64]. miR-141 and miR-200a, through Homebox Distal-Less-5 (Dlx5) decreased the expression, among others, of BMP-2 [68]. Recent studies demonstrated that miR-206 inhibits connexin 43 expression and tend to impair osteoblast differentiation [69]. The miR-23a ~ 27a ~ 24-2 complex inhibits osteoblastogenesis by negative regulation of SATB2 [65]. miR-29a and miR-29c are involved in the regulation of Wnt pathway and inhibit the expression of osteonectin [70]. Finally, miR-125b inhibits Erb-2 and negatively regulates osteoblast proliferation [71]. 

Contrary to the negative role of various miRNAs, it has been described that some miRNAs promote osteogenesis. miR-29b, miR208 and miR-210 modulate BMP/TGF/Activin signaling [72]. miR-218 negatively regulates Erb1 (TOB1) and sclerostin (SOST) [73]. miR-196 targets Hoxc8 (a Smad1 negative regulator) impairing adipogenesis and promoting osteogenesis [74]. Finally, miR-335-5p attenuates Dkk1, an inhibitor of the Wnt pathway, and consequently increase Wnt pathway activity and tends to facilitate osteoblast formation [75]. The exact role of these mechanisms in the pathogenesis of bone disorders remains to be elucidated. Nevertheless, whether they are involved in skeletal disorders or not, those studies suggest that the modulation of epigenetic mechanisms may be used to improve bone tissue engineering and in general in bone regenerative medicine, specifically when there is a need to form new bone to heal a local skeletal defect.

## EPIGENETIC CHANGES DURING OSTEOCLASTOGENESIS: CONTROL OF REMODELING 

Activation of osteoclastogenesis is required for remodeling. Although not definitively proved in humans, some experimental data suggest that osteocytes start the cascade of events for osteoclast differentiation, presumably releasing cytokines and other factors that modulate the activity and differentiation of cells in both the osteoblastic and osteoclastic lineages [35,39]. On the other hand, there is extensive evidence for a role of cells of the osteoblastic lineage in the regulation of osteoclastogenesis. In fact, different molecules produced by osteoblastic cells, including stromal derived factor (SDF), the monocyte chemotactic protein type 1 (MCP-1) or the macrophage colony-stimulating factor (M-CSF), have been shown to either attract osteoclast precursors to the sites of bone remodeling or stimulate their proliferation [76-78]. Other critical factor in osteoclastogenesis is the Receptor activator of nuclear factor Kappa-B ligand (RANKL) which initiates the cascade of events for osteoclast maturation. RANKL is produced by many cell types, such as immune, vascular and stromal cells. However, it is well accepted that osteoblasts and probably osteocytes, are the major sources of this cytokine in bone tissue. Interestingly, Kitazawa *et al*. demonstrated that DNA methylation at the proximal promoter of the RANKL gene inhibits its expression in a murine system, which results in impaired osteoclastogenesis [79]. Our group has recently reported a similar regulation of RANKL expression by the methylation/demethylation of a CpG island located in the vicinity of the RANKL promoter in human bone cells [80]. 

RANKL acts by binding to its receptor RANK located in the membrane of osteoclast precursors [81,82]. This induces a cascade of molecular events that leads to the activation and nuclear translocation of the nuclear factor of activated T cells (NFATc1) [83] which, in turn, induces the expression of a variety of target genes, thus promoting osteoclast differentiation. Recent reports suggest that NFATc1 activity may be controlled by Jumonji, a histone demethylase, as well as by miR-146a [84,85]. 

**Fig. (3):**
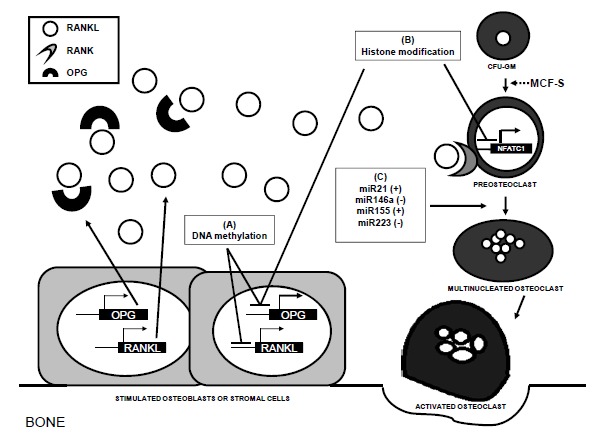
Mechanisms involved in the epigenetic regulation of osteoclastogenesis. **A**) DNA methylation at CpG-rich areas within the RANKL and OPG promoters blocks gene transcription, impairing osteoclastogenesis. **B**) Histone post-translational modifications have been shown to directly modulate NFATc1 activity and OPG transcription. **C**) miRNAs have both positive and negative effects on the progression of osteoclast precursor differentiation. miR-21 and miR-155 have a positive effect by decreasing the levels of inhibitory genes, whereas miR-146a and miR-223 tend to impair osteoclast differentiation.

It is important to note that osteoblasts also synthesize and secrete osteoprotegerin (OPG), a soluble decoy receptor of RANKL that inhibits its interaction with RANK [86,87]. Together with RANKL and RANK, these three factors constitute the RANKL-RANK-OPG system. The RANKL/OPG balance at the bone microenvironment is considered a major determinant of bone mass. Indeed, RANKL and RANK knockout mice show a marked increase in bone mass, mainly due to a decrease in osteoclast numbers, whereas OPG knockout mice show the opposite phenotype [88-90]. Therefore a precise control of the expression of these genes is required for normal bone homeostasis, as well as for bone adaptation to environmental factors. Not only RANKL and NFATc1, but also other genes related to this signaling system are epigenetically regulated (Fig. **3**). Indeed, it has been shown that DNA methylation and histone modifications cooperate to regulate OPG expression in nasopharyngeal carcinomas [91]. Data from our laboratory suggest that the methylation of CpG-rich regions in the OPG gene may also regulate OPG levels in human osteoblastic cells and non-neoplastic bone tissue [80]. Furthermore, results from *in vitro* experiments using histone deacetylase inhibitors suggest that osteoclast activity is modulated by these enzymes [92]. 

Besides DNA methylation and histone post-translational modifications, growing evidence supports the notion that osteoclastogenesis may be regulated by miRNAs, acting in both a positive and negative way (Fig. **3C**). In support of this notion, it has been shown that specific ablation of the Dicer enzyme in osteoclastic cells suppresses bone resorption [93]. Additionally, over-expression of miR-223 inhibits osteoclastogenesis [94], whereas, on the contrary, two miRNAs, miR-21 and miR-155, have been reported to exert a permissive role on osteoclast differentiation, decreasing the levels of some inhibitory genes [95,96]. As an obvious consequence of those studies, it is tempting to speculate that the inhibition of miRNA facilitating osteoclastogenesis might represent an attractive new approach to treat disorders characterized by an accelerated bone resorption. Interestingly, it has been suggested that not only osteoblasts regulate osteoclast activity, but osteoclasts may also, in turn, influence osteoblast differentiation. Several mechanisms may be involved, including changes in the expression levels of some miRNAs [97]. 

## THE ROLE OF EPIGENETIC MARKS IN OSTEOPOROSIS

The role of epigenetics in osteoporosis is just starting to be studied. However, it is gradually being postulated as a key concept, because epigenetic mechanisms are involved in the interactions between the genome and the environment, and, on the other hand, they drive the differentiation programs for cell fate, which, as already mentioned, play a critical role in remodeling of bone tissue. Although the exact relationship between epigenotypes and disease phenotypes is still to be elucidated, it is known that epigenetic marks change during aging, including a global decrease in the abundance of 5- methylcytosines and some histone modifications [98]. Since osteoporosis is an age-related disease, it could be speculated that those age-related changes in epigenetic marks participate in the pathophysiology of the disease. However, at the present time this remains merely speculative.

There is evidence showing that the environment has a strong influence on bone mass, even during the gestation period. Indeed, the intra-uterus environment has been shown to affect the development of the fetal skeleton with effects not only evident at birth, but also persisting later in life (recently reviewed by Holroyd *et al*.) [99]. For instance, the velocity of fetal femur length growth has been associated with the skeletal size at 4 years of age [100]. Maternal factors that have been associated with neonatal bone mass include body weight, fat stores, physical activity and smoking. Likewise, vitamin D and calcium availability during pregnancy have been shown to influence both fetal skeletal development and childhood bone mass [101]. Furthermore, poor growth in uterus and during the first years of life seems to be associated with thinner bones and the risk of fractures in the adulthood [102]. 

Overall, these and other data strongly suggest that, besides genetic factors, environmental influences during the early phases of development and growth influence the peak bone mass, and consequently the risk of osteoporosis. The mechanisms involved remain to be elucidated, but some data indeed suggest that they may include epigenetic changes. Most data come from animal studies. For instance, feeding pregnant rats with a low protein diet resulted in a reduced bone mass in the offspring that persisted up to 75 weeks of age and was accompanied by an impaired proliferation and differentiation of bone marrow stromal cells, a population including osteoblast precursors [103,104]. It has been shown that maternal dietary restriction in rats induces changes in the methylation status of the genes coding for the glucocorticoid receptor and the peroxisomal proliferator-activated receptor alpha (PPARα) which persist after weaning and are transmitted to future generations [105-107]. There is also indirect evidence for an influence of early life conditions on the gene methylation status in humans. Thus, Dutch subjects exposed prenatally to famine in 1944 have been reported to have an increased risk of a variety of metabolic and neurological disorders, along an abnormal methylation status of various gene promoters, including IGF2 and other genes related to tissue growth and metabolism [108,109]. However, a direct proof of a link between in uterus environment, DNA methylation and bone mass in humans is not available yet. 

Besides DNA methylation, the roles of miRNAs and histone modifications in osteoporosis are being actively studied. In a recent report, three DNA polymorphisms have been found to alter the binding affinity of specific miRNAs that regulate the levels of the FGF2 gene and contribute to determine the susceptibility to osteoporosis [110]. A study by Li *et al*. represents an interesting example of interaction between two epigenetic mechanisms, miRNA and histone code. They described a homozygous mutation in pre-miR-2861 that impaired the formation of the mature miRNA and caused a decrease of bone mass, mainly by targeting HDAC5 [111]. Eskildsen *et al*. suggested that miR-138 could be involved in the reduced levels of the focal adhesion kinase gene observed in osteoporotic and osteoarthritic patients [112]. On the other hand, a decreased expression of heparanase (HPSE) has been observed in osteoblasts of osteoporotic patients, in comparison with those isolated from healthy individuals. HPSE is presumably involved in histone phosphorylation [113]. 

Some drugs used or postulated as treatments for osteoporotic patients have been suggested to induce epigenetic changes. This is the hormone, which modulates histone deacetylase activity in osteoblasts [114]. Although not approved as an anti-osteoporotic drug, resveratrol, a polyphenolic phytoestrogen, has been shown to exhibit potent bone anti-catabolic properties, and presumably acts influencing the activity of sirtuin 1, an histone deacetylase [115]. Bisphosphonates are the most widely drugs used to treat osteoporotic patients. They are potent inhibitors of bone resorption by targeting the mevalonate pathway in the osteoclasts. Moreover, some data suggest that they may also have effects on bone formation. In line with this, it has been reported that bisphosphonates modulate the expression of some miRNAs, including miR-18a, miR-133a, miR-141 and miR-19a, that influence osteoblast proliferation [116]. Further studies are needed to elucidate whether it is central to bisphosphonate action or just an epiphenomenon associated with their osteoclast inhibitory effects.

## PERSPECTIVES

As reviewed above, both epidemiological and experimental data suggest that epigenetic mechanisms influence skeletal development and the risk of bone disorders. Nevertheless, further research is required to elucidate the exact role of these mechanisms in the phenotypic changes responsible for skeletal diseases. In particular, the specific changes in DNA methylation, histone modifications and miRNA expression involved in the pathogenesis of osteoporosis remain to be identified. Although animal models provide a useful resource for the investigation of these mechanisms, so far it is unclear to what extent the results can be extrapolated to humans. Therefore, studies in human systems are of special interest. 

Genome-wide studies have identified a number of genes associated with bone mass. However, they only explain a small fraction of the bone mass variation observed in the population [117]. The explanations for this unexpected result are unclear, but may include the interaction between genetic and epigenetic mechanisms. In fact, epigenetic changes regulate gene transcription, but there is also growing evidence for an influence of genetic (i.e., DNA sequence) variants on DNA methylation and miRNA-dependent mechanisms (“genetical epigenomics”) [118].

New technologies such as epigenetic microarrays and ultra-high throughput sequencing may help to establish a complete epigenetic landscape of normal bone and skeletal diseases on a genome-wide basis. For instance, the comparison of cytosine methylation in subjects with different bone phenotypes may lead to the identification of differentially methylated regions involved in disease pathogenesis. It is worth mentioning that those studies present more difficulties than genome studies, due to the cell/tissue specificity of epigenetic marks and their instability over time. Since bone is a very heterogeneous tissue, composed of a variety of cells in bone itself and bone marrow, the results obtained in bone tissue samples may be hard to interpret. Nevertheless, the identification of disease-specific patterns of DNA methylation or miRNA expression may give valuable information. First, it may help to identify genes and metabolic pathways involved in the disease and consequently lead to discover new targets for disease therapy. On the other hand, the identification of disease-specific epigenetic marks may be helpful for both diagnosis and prevention. DNA, either methylated or unmethylated, and miRNA are more stable than RNA [119], which may be an advantage for clinical use. Of course, to this respect, markers in accessible samples, especially those present in circulating blood and body fluids, will be much more feasible to be used as diagnostic tools [120].

Studies of bone epigenetics may not only point to certain genes as new targets for therapy, but may represent the foundation of new therapies based on the control of epigenetic mechanisms. Some demethylating agents are already used to treat neoplastic disorders [121,122]. It would be interesting to study their effects on “bone genes”. However, their potential utility is limited by their widespread effects. Agents able to modulate specifically the epigenetic control of genes in a given pathway would be much more useful. In line with this, therapies based on small RNAs, mimicking or interfering with miRNAs, might be more specific and promising [123,124]. Both DNA methylation- and miRNA-based treatments may be also useful in regenerative medicine. For instance, one could envision a future in which the demethylation of the promoters of certain genes driving osteoblast proliferation in a limited region of the skeleton might be used to fill bone defects and enhance the consolidation of delayed-union fractures.

Another priority research line in this field may be the study of the potential influence of current therapies on the epigenetic regulation of bone factors. Of particular interest, the effect of anabolic agents, such as parathyroid hormone and bone morphogenetic proteins, on the methylation of CpG-rich regions of genes involved in the regulation of bone remodeling should be investigated. If these drugs indeed modify DNA methylation patterns, prior knowledge of the patient methylation profile might help to individualize therapy according to his epigenome (“pharmacoepigenomics”).

Overall, although data are still scarce, studies highlighted in this review invite to think that epigenetics will be an important subject in the bone research field in the next years.

## Figures and Tables

**Table 1.** Some Studies Identifying miRNAs Involved in the Regulation of Osteoblast Differentiation

miR # ID	Observation	Ref.
miR-125b	Inhibits proliferation and impairs osteoblast differentiation	[71]
miR-133/135-a	Target Runx2 and Smad 5, impairing osteoblast differentiation	[64]
miR-135b	Targets sialoprotein, osterix, osteocalcin and Runx2	[63]
miR-141/200a	Target Dlx5, impairing osteoblast differentiation	[68]
miR-196a	Target Hoxc8, enhancing osteogenic differentiation	[74]
miR-204/211	Target Runx2 impairing osteoblast differentiation	[62]
miR-206	Targets connexin 43, impairs osteoblast differentiation	[69]
miR-208	Indirectly upregulates BMPs	[125]
miR-210	Targets AcvR1b, inducing osteogenesis.	[126]
miR-218	Decreases SOST and TOB1 expression	[73]
miR-23a/27a/24-2	Regulated by Runx2; target SATB2 and Runx2.	[65]
miR-26a	Targets Smad1, impairing osteoblast differentiation	[67]
miR-2861	Targets HDAC5, inducing osteogenic differentiation	[111]
miR-29/29c	Modulate Wnt pathway; target osteonectin	[70]
miR-29b	Promotes osteogenic differentiation	[72]
miR-30c/34c/133a/135a/137/204/205/217/338	Target Runx2 and impair osteoblast differentiation	[127]
miR-31/106a/148a/424/30c/15b	Differentially expressed during MSC osteogenic differentiation	[59]
miR-335-5p	Negative regulation of DKK1, increasing Wnt signaling	[75]

## References

[1] Melton LJ (1995). How many women have osteoporosis now?. J. Bone Miner. Res.

[2] Ralston SH (2002). Genetic control of susceptibility to osteoporosis. J. Clin. Endocrinol. Metab.

[3] Peacock M, Turner CH, Econs MJ, Foroud T (2002). Genetics of osteoporosis. Endocr. Rev.

[4] Nguyen TV, Howard GM, Kelly PJ, Eisman JA (1998). Bone mass, lean mass, and fat mass: same genes or same environments?. Am. J. Epidemiol.

[5] Cummings SR, Nevitt MC, Browner WS, Stone K, Fox KM, Ensrud KE, Cauley J, Black D, Vogt TM (1995). Risk factors for hip fracture in white women. N. Engl. J. Med.

[6] Kanis JA, Johansson H, Oden A, Johnell O, De Laet C, Eisman JA, McCloskey EV, Mellstrom D, Melton LJ, Pols HA, Reeve J, Silman AJ, Tenenhouse A (2004). A family history of fracture and fracture risk: a meta-analysis. Bone.

[7] Hernandez CJ, Beaupr‚ GS, Carter DR (2003). A theoretical analysis of the relative influences of peak BMD, age-related bone loss and menopause on the development of osteoporosis. Osteoporos. Int.

[8] Brown LB, Streeten EA, Shapiro JR, McBride D, Shuldiner AR, Peyser PA, Mitchell BD (2005). Genetic and environmental influences on bone mineral density in pre- and post-menopausal women. Osteoporos. Int.

[9] Michaelsson K, Melhus H, Ferm H, Ahlbom A, Pedersen NL (2005). Genetic liability to fractures in the elderly. Arch. Intern. Med.

[10] Ralston SH, de Crombrugghe B (2006). Genetic regulation of bone mass and susceptibility to osteoporosis. Genes Dev.

[11] Velasco J, Zarrabeitia MT, Prieto JR, Perez-Castrillon JL, Perez-Aguilar MD, Perez-Nunez MI, Sanudo C, Hernandez-Elena J, Calvo I, Ortiz F, Gonzalez-Macias J, Riancho JA (2010). Wnt pathway genes in osteoporosis and osteoarthritis: differential expression and genetic association study. Osteoporos. Int.

[12] Esteller M (2011). Cancer Epigenetics for the 21st Century: What's Next?. Genes Cancer.

[13] Irizarry RA, Ladd-Acosta C, Wen B, Wu Z, Montano C, Onyango P, Cui H, Gabo K, Rongione M, Webster M, Ji H, Potash JB, Sabunciyan S, Feinberg AP (2009). The human colon cancer methylome shows similar hypo- and hypermethylation at conserved tissue-specific CpG island shores. Nat. Genet.

[14] Munzel M, Globisch D, Carell T (2011). 5-Hydroxymethylcytosine, the sixth base of the genome. Angew. Chem. Int. Ed Engl.

[15] Hermann A, Gowher H, Jeltsch A (2004). Biochemistry and biology of mammalian DNA methyltransferases. Cell Mol. Life Sci.

[16] Turek-Plewa J, Jagodzinski PP (2005). The role of mammalian DNA methyltransferases in the regulation of gene expression. Cell Mol. Biol. Lett.

[17] Kouzarides T (2007). Chromatin modifications and their function. Cell.

[18] Shilatifard A (2006). Chromatin modifications by methylation and ubiquitination: implications in the regulation of gene expression. Annu. Rev. Biochem.

[19] Santos-Rosa H, Caldas C (2005). Chromatin modifier enzymes, the histone code and cancer. Eur. J. Cancer.

[20] Lee RC, Feinbaum RL, Ambros V (1993). The C. elegans heterochronic gene lin-4 encodes small RNAs with antisense complementarity to lin-14. Cell.

[21] Bentwich I, Avniel A, Karov Y, Aharonov R, Gilad S, Barad O, Barzilai A, Einat P, Einav U, Meiri E, Sharon E, Spector Y, Bentwich Z (2005). Identification of hundreds of conserved and nonconserved human microRNAs. Nat. Genet.

[22] Friedman RC, Farh KK, Burge CB, Bartel DP (2009). Most mammalian mRNAs are conserved targets of microRNAs. Genome Res.

[23] Kim VN (2005). MicroRNA biogenesis: coordinated cropping and dicing. Nat. Rev. Mol. Cell Biol.

[24] Bartel DP (2004). MicroRNAs: genomics, biogenesis, mechanism, and function. Cell.

[25] Fuks F, Hurd PJ, Wolf D, Nan X, Bird AP, Kouzarides T (2003). The methyl-CpG-binding protein MeCP2 links DNA methylation to histone methylation. J. Biol. Chem.

[26] Gal-Yam EN, Saito Y, Egger G, Jones PA (2008). Cancer epigenetics: modifications, screening, and therapy. Annu. Rev. Med.

[27] Szulwach KE, Li X, Smrt RD, Li Y, Luo Y, Lin L, Santistevan NJ, Li W, Zhao X, Jin P (2010). Cross talk between microRNA and epigenetic regulation in adult neurogenesis. J. Cell Biol.

[28] Sato F, Tsuchiya S, Meltzer SJ, Shimizu K (2011). MicroRNAs and epigenetics. FEBS J.

[29] Liang R, Bates DJ, Wang E (2009). Epigenetic Control of MicroRNA Expression and Aging. Curr. Genomics.

[30] Teti A (2011). Bone Development: Overview of Bone Cells and Signaling. Curr. Osteoporos. Rep.

[31] Nakashima T, Hayashi M, Fukunaga T, Kurata K, Oh-Hora M, Feng JQ, Bonewald LF, Kodama T, Wutz A, Wagner EF, Penninger JM, Takayanagi H (2011). Evidence for osteocyte regulation of bone homeostasis through RANKL expression. Nat. Med.

[32] Xiong J, Onal M, Jilka RL, Weinstein RS, Manolagas SC, O'Brien CA (2011). Matrix-embedded cells control osteoclast formation. Nat. Med.

[33] Logar DB, Komadina R, Prezelj J, Ostanek B, Trost Z, Marc J (2007). Expression of bone resorption genes in osteoarthritis and in osteoporosis. J. Bone Miner. Metab.

[34] D'Amelio P, Roato I, D'Amico L, Veneziano L, Suman E, Sassi F, Bisignano G, Ferracini R, Gargiulo G, Castoldi F, Pescarmona GP, Isaia GC (2011). Bone and bone marrow pro-osteoclastogenic cytokines are up-regulated in osteoporosis fragility fractures. Osteoporos. Int.

[35] Rochefort GY, Pallu S, Benhamou CL (2010). Osteocyte: the unrecognized side of bone tissue. Osteoporos. Int.

[36] Jackson L, Jones DR, Scotting P, Sottile V (2007). Adult mesenchymal stem cells: differentiation potential and therapeutic applications. J. Postgrad. Med.

[37] Noble BS (2008). The osteocyte lineage. Arch. Biochem. Biophys.

[38] Kang MI, Kim HS, Jung YC, Kim YH, Hong SJ, Kim MK, Baek KH, Kim CC, Rhyu MG (2007). Transitional CpG methylation between promoters and retroelements of tissue-specific genes during human mesenchymal cell differentiation. J. Cell Biochem.

[39] Bonewald LF (2011). The amazing osteocyte. J. Bone Miner. Res.

[40] Hantusch B, Kalt R, Krieger S, Puri C, Kerjaschki D (2007). Sp1/Sp3 and DNA-methylation contribute to basal transcriptional activation of human podoplanin in MG63 versus Saos-2 osteoblastic cells. BMC. Mol. Biol.

[41] Miao D, Scutt A (2002). Histochemical localization of alkaline phosphatase activity in decalcified bone and cartilage. J. Histochem. Cytochem.

[42] Delgado-Calle J, Sanudo C, Sanchez-Verde L, Garcia-Renedo RJ, Arozamena J, Riancho JA (2011). Epigenetic regulation of alkaline phosphatase in human cells of the osteoblastic lineage. Bone.

[43] Semenov M, Tamai K, He X (2005). SOST is a ligand for LRP5/LRP6 and a Wnt signaling inhibitor. J. Biol. Chem.

[44] Delgado-Calle J, Sanudo C, Bolado A, Fernández AF, Arozamena J, Pascual-Carra MA, Rodriguez-Rey JC, Fraga M F, Bonewald LF, Riancho JA (2011). DNA methylation contributes to the regulation of sclerostin expression in human osteocytes. J. Bone Miner. Res.

[45] Cohen-Kfir E, Artsi H, Levin A, Abramowitz E, Bajayo A, Gurt I, Zhong L, D'Urso A, Toiber D, Mostoslavsky R, Dresner-Pollak R (2011). Sirt1 Is a Regulator of Bone Mass and a Repressor of Sost Encoding for Sclerostin: A Bone Formation Inhibitor. Endocrinology.

[46] Locklin RM, Oreffo RO, Triffitt JT (1998). Modulation of osteogenic differentiation in human skeletal cells *in vitro* by 5-azacytidine. Cell Biol. Int.

[47] Arnsdorf EJ, Tummala P, Castillo AB, Zhang F, Jacobs CR (2010). The epigenetic mechanism of mechanically induced osteogenic differentiation. J. Biomech.

[48] Villagra A, Gutierrez J, Paredes R, Sierra J, Puchi M, Imschenetzky M, Wijnen AA, Lian J, Stein G, Stein J, Montecino M (2002). Reduced CpG methylation is associated with transcriptional activation of the bone-specific rat osteocalcin gene in osteoblasts. J. Cell Biochem.

[49] Dansranjavin T, Krehl S, Mueller T, Mueller LP, Schmoll HJ, Dammann RH (2009). The role of promoter CpG methylation in the epigenetic control of stem cell related genes during differentiation. Cell Cycle.

[50] Loeser RF, Im HJ, Richardson B, Lu Q, Chubinskaya S (2008). Methylation of the OP-1 promoter: potential role in the age-related decline in OP-1 expression in cartilage. Osteoarthritis Cartilage.

[51] Lee JY, Lee YM, Kim MJ, Choi JY, Park EK, Kim SY, Lee SP, Yang JS, Kim DS (2006). Methylation of the mouse DIx5 and Osx gene promoters regulates cell type-specific gene expression. Mol. Cell.

[52] Penolazzi L, Lambertini E, Giordano S, Sollazzo V, Traina G, del Senno L, Piva R (2004). Methylation analysis of the promoter F of estrogen receptor alpha gene: effects on the level of transcription on human osteoblastic cells. J. Steroid Biochem. Mol. Biol.

[53] Demura M, Bulun SE (2008). CpG dinucleotide methylation of the
CYP19 I.3/II promoter modulates cAMP-stimulated aromatase
activity. Mol. Cell Endocrinol.

[54] Gordon JA, Hassan MQ, Koss M, Montecino M, Selleri L, van Wijnen AJ, Stein JL, Stein GS, Lian JB (2011). Epigenetic regulation of early osteogenesis and mineralized tissue formation by a HOXA10-PBX1-associated complex. Cells Tissues Organs.

[55] Shen J, Hovhannisyan H, Lian JB, Montecino MA, Stein GS, Stein JL, van Wijnen AJ (2003). Transcriptional induction of the osteocalcin gene during osteoblast differentiation involves acetylation of histones h3 and h4. Mol. Endocrinol.

[56] Hassan MQ, Saini S, Gordon JA, van Wijnen AJ, Montecino M, Stein JL, Stein GS, Lian JB (2009). Molecular switches involving homeodomain proteins, HOXA10 and RUNX2 regulate osteoblastogenesis. Cells Tissues Organs.

[57] Fan Z, Yamaza T, Lee JS, Yu J, Wang S, Fan G, Shi S, Wang CY (2009). BCOR regulates mesenchymal stem cell function by epigenetic mechanisms. Nat. Cell Biol.

[58] Jensen ED, Gopalakrishnan R, Westendorf JJ (2010). Regulation of gene expression in osteoblasts. Biofactors.

[59] Gao J, Yang T, Han J, Yan K, Qiu X, Zhou Y, Fan Q, Ma B (2011). MicroRNA expression during osteogenic differentiation of human multipotent mesenchymal stromal cells from bone marrow. J. Cell Biochem.

[60] Komori T (2010). Regulation of osteoblast differentiation by Runx2. Adv. Exp. Med. Biol.

[61] Sevetson B, Taylor S, Pan Y (2004). Cbfa1/RUNX2 directs specific expression of the sclerosteosis gene (SOST). J. Biol. Chem.

[62] Huang J, Zhao L, Xing L, Chen D (2010). MicroRNA-204 regulates Runx2 protein expression and mesenchymal progenitor cell differentiation. Stem Cell.

[63] Schaap-Oziemlak AM, Raymakers RA, Bergevoet SM, Gilissen C, Jansen BJ, Adema GJ, Kogler G, le Sage C, Agami R, van der Reijden BA, Jansen JH (2010). MicroRNA hsamiR-135b regulates mineralization in osteogenic differentiation of human unrestricted somatic stem cells. Stem Cells Dev.

[64] Li Z, Hassan MQ, Volinia S, van Wijnen AJ, Stein JL, Croce CM, Lian JB, Stein GS (2008). A microRNA signature for a BMP2-induced osteoblast lineage commitment program. Proc. Natl. Acad. Sci. U. S. A.

[65] Hassan MQ, Gordon JA, Beloti MM, Croce CM, van Wijnen AJ, Stein JL, Stein GS, Lian JB (2010). A network connecting Runx2, SATB2, and the miR-23a~27a~24-2 cluster regulates the osteoblast differentiation program. Proc. Natl. Acad. Sci. U. S. A.

[66] Javed A, Bae JS, Afzal F, Gutierrez S, Pratap J, Zaidi SK, Lou Y, van Wijnen AJ, Stein JL, Stein GS, Lian JB (2008). Structural coupling of Smad and Runx2 for execution of the BMP2 osteogenic signal. J. Biol. Chem.

[67] Luzi E, Marini F, Sala SC, Tognarini I, Galli G, Brandi ML (2008). Osteogenic differentiation of human adipose tissue-derived stem cells is modulated by the miR-26a targeting of the SMAD1 transcription factor. J. Bone Miner. Res.

[68] Itoh T, Nozawa Y, Akao Y (2009). MicroRNA-141 and -200a are involved in bone morphogenetic protein-2-induced mouse pre-osteoblast differentiation by targeting distal-less homeobox 5. J. Biol. Chem.

[69] Inose H, Ochi H, Kimura A, Fujita K, Xu R, Sato S, Iwasaki M, Sunamura S, Takeuchi Y, Fukumoto S, Saito K, Nakamura T, Siomi H, Ito H, Arai Y, Shinomiya K, Takeda S (2009). A microRNA regulatory mechanism of osteoblast differentiation. Proc. Natl. Acad. Sci. U. S. A.

[70] Kapinas K, Kessler C, Ricks T, Gronowicz G, Delany AM (2010). miR-29 modulates Wnt signaling in human osteoblasts through a positive feedback loop. J. Biol. Chem.

[71] Mizuno Y, Yagi K, Tokuzawa Y, Kanesaki-Yatsuka Y, Suda T, Katagiri T, Fukuda T, Maruyama M, Okuda A, Amemiya T, Kondoh Y, Tashiro H, Okazaki Y (2008). miR-125b inhibits osteoblastic differentiation by down-regulation of cell proliferation. Biochem. Biophys. Res. Commun.

[72] Li Z, Hassan MQ, Jafferji M, Aqeilan RI, Garzon R, Croce CM, van Wijnen AJ, Stein JL, Stein GS, Lian JB (2009). Biological functions of miR-29b contribute to positive regulation of osteoblast differentiation. J. Biol. Chem.

[73] Jafferji M (2009). Regulation of osteoblast differentiation by microRNAs. PhD Thesis, Worcester Polytechnic Institute: Worcester, MA.

[74] Kim YJ, Bae SW, Yu SS, Bae YC, Jung JS (2009). miR-196a regulates proliferation and osteogenic differentiation in mesenchymal stem cells derived from human adipose tissue. J. Bone Miner. Res.

[75] Zhang J, Tu Q, Bonewald LF, He X, Stein G, Lian J, Chen J (2011). Effects of miR-335-5p in modulating osteogenic differentiation by specifically downregulating Wnt antagonist DKK1. J. Bone Miner. Res.

[76] Sims NA, Gooi JH (2008). Bone remodeling: Multiple cellular interactions required for coupling of bone formation and resorption. Semin. Cell Dev. Biol.

[77] Raggatt LJ, Partridge NC (2010). Cellular and molecular mechanisms of bone remodeling. J. Biol. Chem.

[78] Matsuo K, Irie N (2008). Osteoclast-osteoblast communication. Arch. Biochem. Biophys.

[79] Kitazawa R, Kitazawa S (2007). Methylation status of a single CpG locus 3 bases upstream of TATA-box of receptor activator of nuclear factor-kappaB ligand (RANKL) gene promoter modulates cell- and tissue-specific RANKL expression and osteoclastogenesis. Mol. Endocrinol.

[80] Delgado-Calle J, Sañudo C, Fernández AF, García-Renedo R, Fraga MF, Riancho JA (2011). Epigenetics mechanisms modulate the expression of genes controlling osteoclast differentiation. J Bone Miner. Res.

[81] Kearns AE, Khosla S, Kostenuik PJ (2008). Receptor activator of nuclear factor kappaB ligand and osteoprotegerin regulation of bone remodeling in health and disease. Endocr. Rev.

[82] Wright HL, McCarthy HS, Middleton J, Marshall MJ (2009). RANK, RANKL and osteoprotegerin in bone biology and disease. Curr. Rev. Musculoskelet. Med.

[83] Zhao Q, Wang X, Liu Y, He A, Jia R (2010). NFATc1: functions in osteoclasts. Int J Biochem Cell Biol.

[84] Yasui T, Hirose J, Tsutsumi S, Nakamura K, Aburatani H, Tanaka S (2011). Epigenetic regulation of osteoclast differentiation: Possible involvement of Jmjd3 in the histone demethylation of Nfatc1. J. Bone Miner. Res.

[85] Nakasa T, Shibuya H, Nagata Y, Niimoto T, Ochi M (2011). The inhibitory effect of microRNA-146a expression on bone destruction in collagen-induced arthritis. Arthritis Rheum.

[86] Boyce BF, Xing L (2008). Functions of RANKL/RANK/OPG in bone modeling and remodeling. Arch. Biochem. Biophys.

[87] Aoki S, Honma M, Kariya Y, Nakamichi Y, Ninomiya T, Takahashi N, Udagawa N, Suzuki H (2010). Function of OPG as a traffic regulator for RANKL is crucial for controlled osteoclastogenesis. J. Bone Miner. Res.

[88] Kong YY, Yoshida H, Sarosi I, Tan HL, Timms E, Capparelli C, Morony S, Oliveira-dos-Santos AJ, Van G, Itie A, Khoo W, Wakeham A, Dunstan CR, Lacey DL, Mak TW, Boyle WJ, Penninger JM (1999). OPGL is a key regulator of osteoclastogenesis, lymphocyte development and lymph-node organogenesis. Nature.

[89] Bucay N, Sarosi I, Dunstan CR, Morony S, Tarpley J, Capparelli C, Scully S, Tan HL, Xu W, Lacey DL, Boyle WJ, Simonet WS (1998). osteoprotegerin-deficient mice develop early onset osteoporosis and arterial calcification. Genes Dev.

[90] Li J, Sarosi I, Yan XQ, Morony S, Capparelli C, Tan HL, McCabe S, Elliott R, Scully S, Van G, Kaufman S, Juan SC, Sun Y, Tarpley J, Martin L, Christensen K, McCabe J, Kostenuik P, Hsu H, Fletcher F, Dunstan CR, Lacey DL, Boyle WJ (2000). RANK is the intrinsic hematopoietic cell surface receptor that controls osteoclastogenesis and regulation of bone mass and calcium metabolism. Proc. Natl. Acad. Sci. U. S. A.

[91] Lu TY, Kao CF, Lin CT, Huang DY, Chiu CY, Huang YS, Wu HC (2009). DNA methylation and histone modification regulate silencing of OPG during tumor progression. J. Cell Biochem.

[92] Cantley MD, Fairlie DP, Bartold PM, Rainsford KD, Le GT, Lucke AJ, Holding CA, Haynes DR (2011). Inhibitors of histone deacetylases in class I and class II suppress human osteoclasts *in vitro*. J. Cell Physiol.

[93] Mizoguchi F, Izu Y, Hayata T, Hemmi H, Nakashima K, Nakamura T, Kato S, Miyasaka N, Ezura Y, Noda M (2010). Osteoclast-specific Dicer gene deficiency suppresses osteoclastic bone resorption. J. Cell Biochem.

[94] Sugatani T, Hruska KA (2007). MicroRNA-223 is a key factor in osteoclast differentiation. J. Cell Biochem.

[95] Mann M, Barad O, Agami R, Geiger B, Hornstein E (2010). miRNA-based mechanism for the commitment of multipotent progenitors to a single cellular fate. Proc. Natl. Acad. Sci. U. S. A.

[96] Sugatani T, Vacher J, Hruska KA (2011). A microRNA expression signature of osteoclastogenesis. Blood.

[97] Yu F, Cui Y, Zhou X, Zhang X, Han J (2011). Osteogenic differentiation of human ligament fibroblasts induced by conditioned medium of osteoclast-like cells. Biosci. Trends.

[98] Fraga MF, Esteller M (2007). Epigenetics and aging: the targets and the marks. Trends Genet.

[99] Holroyd C, Harvey N, Dennison E, Cooper C (2011). Epigenetic influences in the developmental origins of osteoporosis. Osteoporos. Int.

[100] Harvey N, Mahon P, Robinson S, Nisbet C, Javaid M, Crozier S, Inskip H, Godfrey K, Arden N, Dennison E, Cooper C (2009). Different Indices of Fetal Growth Predict Bone Size and Volumetric Density at 4 Years Old. J Bone Miner. Res.

[101] Mahon P, Harvey N, Crozier S, Inskip H, Robinson S, Arden N, Swaminathan R, Cooper C, Godfrey K (2010). Low maternal vitamin D status and fetal bone development: cohort study. J Bone Miner. Res.

[102] Baird J, Kurshid MA, Kim M, Harvey N, Dennison E, Cooper C (2011). Does birthweight predict bone mass in adulthood? A systematic review and meta-analysis. Osteoporos. Int.

[103] Lanham SA, Roberts C, Perry MJ, Cooper C, Oreffo RO (2008). Intrauterine programming of bone. Part 2: alteration of skeletal structure. Osteoporos. Int.

[104] Oreffo RO, Lashbrooke B, Roach HI, Clarke NM, Cooper C (2003). Maternal protein deficiency affects mesenchymal stem cell activity in the developing offspring. Bone.

[105] Lillycrop KA, Phillips ES, Torrens C, Hanson MA, Jackson AA, Burdge GC (2008). Feeding pregnant rats a protein-restricted diet persistently alters the methylation of specific cytosines in the hepatic PPAR alpha promoter of the offspring. Br. J. Nutr.

[106] Lillycrop KA, Slater-Jefferies JL, Hanson MA, Godfrey KM, Jackson AA, Burdge GC (2007). Induction of altered epigenetic regulation of the hepatic glucocorticoid receptor in the offspring of rats fed a protein-restricted diet during pregnancy suggests that reduced DNA methyltransferase-1 expression is involved in impaired DNA methylation and changes in histone modifications. Br. J. Nutr.

[107] Lillycrop KA, Phillips ES, Jackson AA, Hanson MA, Burdge GC (2005). Dietary protein restriction of pregnant rats induces and folic acid supplementation prevents epigenetic modification of hepatic gene expression in the offspring. J. Nutr.

[108] Heijmans BT, Tobi EW, Stein AD, Putter H, Blauw GJ, Susser ES, Slagboom PE, Lumey LH (2008). Persistent epigenetic differences associated with prenatal exposure to famine in humans. Proc. Natl. Acad. Sci U. S. A.

[109] Heijmans BT, Tobi EW, Lumey LH, Slagboom PE (2009). The epigenome: archive of the prenatal environment. Epigenetics.

[110] Lei SF, Papasian CJ, Deng HW (2011). Polymorphisms in predicted miRNA binding sites and osteoporosis. J. Bone Miner. Res.

[111] Li H, Xie H, Liu W, Hu R, Huang B, Tan YF, Xu K, Sheng ZF, Zhou HD, Wu XP, Luo XH (2009). A novel microRNA targeting HDAC5 regulates osteoblast differentiation in mice and contributes to primary osteoporosis in humans. J. Clin. Invest.

[112] Eskildsen T, Taipaleenmaki H, Stenvang J, Abdallah BM, Ditzel N, Nossent AY, Bak M, Kauppinen S, Kassem M (2011). MicroRNA-138 regulates osteogenic differentiation of human stromal (mesenchymal) stem cells *in vivo*. Proc. Natl. Acad. Sci. U. S. A.

[113] Smith PN, Freeman C, Yu D, Chen M, Gatenby PA, Parish CR, Li RW (2010). Heparanase in primary human osteoblasts. J. Orthop. Res.

[114] Shimizu E, Selvamurugan N, Westendorf JJ, Partridge NC (2007). Parathyroid hormone regulates histone deacetylases in osteoblasts. Ann. N. Y. Acad. Sci.

[115] Tseng PC, Hou SM, Chen RJ, Peng HW, Hsieh CF, Kuo ML, Yen ML (2011). Resveratrol promotes osteogenesis of human mesenchymal stem cells by upregulating RUNX2 gene expression *via* the SIRT1/FOXO3A axis. J. Bone Miner. Res.

[116] Zhou Q, Zhao ZN, Cheng JT, Zhang B, Xu J, Huang F, Zhao RN, Chen YJ (2011). Ibandronate promotes osteogenic differentiation of periodontal ligament stem cells by regulating the expression of microRNAs. Biochem. Biophys. Res. Commun.

[117] Riancho JA (2010). Genetics of osteoporosis: half-full or half-empty?. Crit. Rev. Bone Miner. Metab.

[118] Bell JT, Pai AA, Pickrell JK, Gaffney DJ, Pique-Regi R, Degner JF, Gilad Y, Pritchard JK (2011). DNA methylation patterns associate with genetic and gene expression variation in HapMap cell lines. Genome Biol.

[119] Scholer N, Langer C, Dohner H, Buske C, Kuchenbauer F (2010). Serum microRNAs as a novel class of biomarkers: a comprehensive review of the literature. Exp. Hematol.

[120] Soifer HS, Rossi JJ, Saetrom P (2007). MicroRNAs in disease and potential therapeutic applications. Mol. Ther.

[121] La Thangue NB (2004). Histone deacetylase inhibitors and cancer therapy. J. Chemother.

[122] Santos FP, Kantarjian H, Garcia-Manero G, Issa JP, Ravandi F (2010). Decitabine in the treatment of myelodysplastic syndromes. Expert. Rev. Anticancer Ther.

[123] Fabbri E, Brognara E, Borgatti M, Lampronti I, Finotti A, Bianchi N, Sforza S, Tedeschi T, Manicardi A, Marchelli R, Corradini R, Gambari R (2011). miRNA therapeutics: delivery and biological activity of peptide nucleic acids targeting miRNAs. Epigenomics.

[124] Krutzfeldt J, Rajewsky N, Braich R, Rajeev KG, Tuschl T, Manoharan M, Stoffel M (2005). Silencing of microRNAs *in vivo* with 'antagomirs'. Nature.

[125] Itoh T, Takeda S, Akao Y (2010). MicroRNA-208 modulates BMP-2-stimulated mouse preosteoblast differentiation by directly targeting V-ets erythroblastosis virus E26 oncogene homolog 1. J. Biol. Chem.

[126] Mizuno Y, Tokuzawa Y, Ninomiya Y, Yagi K, Yatsuka-Kanesaki Y, Suda T, Fukuda T, Katagiri T, Kondoh Y, Amemiya T, Tashiro H, Okazaki Y (2009). miR-210 promotes osteoblastic differentiation through inhibition of AcvR1b. FEBS Lett.

[127] Zhang Y, Xie RL, Croce CM, Stein JL, Lian JB, van Wijnen AJ, Stein GS (2011). A program of microRNAs controls osteogenic lineage progression by targeting transcription factor Runx2. Proc. Natl. Acad. Sci. U. S. A.

